# Characterization of a novel 8*R*,11*S*-linoleate diol synthase from *Penicillium chrysogenum* by identification of its enzymatic products[S]

**DOI:** 10.1194/jlr.M061341

**Published:** 2016-02

**Authors:** Kyung-Chul Shin, Min-Ju Seo, Deok-Kun Oh

**Affiliations:** Department of Bioscience and Biotechnology, Konkuk University, Seoul 143-701, Republic of Korea

**Keywords:** 8*R*,11*S*-dihydroxy-9,12(*Z,Z*)-octadecadienoic acid, hydroxy fatty acid, precocious sexual inducer factor

## Abstract

To identify novel fatty acid diol synthases, putative candidate sequences from *Penicillium* species were analyzed, and hydroxy fatty acid production by crude *Penicillium* enzyme extracts was assessed. *Penicillium chrysogenum* was found to produce an unknown dihydroxy fatty acid, a candidate gene implicated in this production was cloned and expressed, and the expressed enzyme was purified. The product obtained by the reaction of the purified enzyme with linoleic acid was identified as 8*R*,11*S*-dihydroxy-9,12(*Z,Z*)-octadecadienoic acid (8*R*,11*S*-DiHODE). The catalytic efficiency of this enzyme toward linoleic acid was the highest among the unsaturated fatty acids tested, indicating that this enzyme was a novel 8*R*,11*S*-linoleate diol synthase (8*R*,11*S*-LDS). A sexual stage in the life cycle of *P. chrysogenum* has recently been discovered, and 8*R*,11*S*-DiHODE produced by 8*R*,11*S*-LDS may constitute a precocious sexual inducer factor, responsible for regulating the sexual and asexual cycles of this fungus.

Sexual and asexual life cycles are controlled by precocious sexual inducer (psi) factors (1), produced by psi-producing oxygenases (Ppo enzymes) in diverse fungi (2–6). Ppo enzymes include PpoA, PpoB, and PpoC. PpoA enzymes (5,8-diol synthases) from *Aspergillus* species, including *Aspergillus clavatus* (5), *Aspergillus fumigatus* (7), *Aspergillus nidulans* (2), *Aspergillus niger* (8), and *Aspergillus terreus* (9), have been shown to convert linoleic acid to 5*S*,8*R*-dihydroxy-9,12(*Z,Z*)-octadecadienoic acid (5*S*,8*R*-DiHODE). PpoB generates compounds with a hydroxyl group at the C8 position of an unsaturated fatty acid, for example, 8*R*-hydroxy-9,12(*Z*,*Z*)-octadecadienoic acid (8*R*-HODE), and PpoC gives rise to compounds with a hydroxyl group at the C10 position, for example, 10*R*-hydroxy-8,12(*E*,*Z*)-octadecadienoic acid (10*R*-HODE) (1–3, 6).

*A. nidulans* is a sexual fungus (2), whereas *A. niger* and *A. clavatus* are known as asexual fungi (8, 10). Although *A. fumigatus* was proposed as an asexual fungus, its sexual cycle was discovered (11). *A. fumigatus*, *A. niger*, and *A. clavatus* convert linoleic acid to not only 5*S*,8*R*-DiHODE but also the by-product 8*R*,11*S*-dihydroxy-9,12(*Z,Z*)-octadecadienoic acid (8*R*,11*S*-DiHODE) (5, 7, 8, 12). *Agaricus bisporus* converts linoleic acid to 8*R*,11*S*-DiHODE (13); however, involvement of an 8,11-diol synthase for 8*R*,11*S*-DiHODE production was speculated but not proved. *Penicillium* species were thought to reproduce exclusively asexually. However, sexual reproduction has recently been demonstrated in *Penicillium roqueforti* and *Penicillium chrysogenum* (14, 15). Exceptionally, *A. bisporus* was well known as a sexual fungus with sexual reproduction and multicellular fruiting bodies (13). 5*S*,8*R*-DiHODE has induced sexual production in fungi (16, 17), while 8*R*,11*S*-DiHODE, suggested as product of PpoB, has been mostly found in asexual fungi except for *A. bisporus*. Therefore, 8*R*,11*S*-DiHODE may be involved in the stimulation of asexual sporulation. However, the biological function of 8*R*,11*S*-DiHODE has not yet been clearly established. Moreover, researches on the psi factors present in *Penicillium* species have not been carried out, as they are yet to be identified in these organisms.

Dihydroxy fatty acids are converted from unsaturated fatty acids by fatty acid diol synthases, including Ppo enzymes. Fatty acid diol synthases are fusion proteins containing N-terminal fatty acid-heme peroxidase [dioxygenase (DOX)] and C-terminal cytochrome P450-heme thiolate (hydroperoxide isomerase) domains. N-terminal DOX domain converts unsaturated fatty acids to hydroperoxy fatty acids, including 8*R*-hydroperoxy-9,12(*Z*,*Z*)-octadecadienoic acid (8*R*-HPODE), which are subsequently isomerized to dihydroxy unsaturated fatty acids, such as 5*S*,8*R*-DiHODE, 7*S*,8*S*-dihydroxy-9,12(*Z*,*Z*)-octadecadienoic acid (7*S*,8*S*-DiHODE), and 8*R*,11*S*-DiHODE by the hydroperoxide isomerase activity of the C-terminal domain, which demonstrates different position specificities. The biochemical properties of 5*S*,8*R*- and 7*S*,8*S*-diol synthases have been characterized previously (18). However, 8*R*,11*S*-diol synthase, which converts linoleic acid to 8*R*,11*S*-DiHODE, has not yet been described.

In the present study, we isolated and characterized 8*R*,11*S*-linoleate diol synthase (8*R*,11*S*-LDS) from *P. chrysogenum* for the first time by assessing the hydroxy fatty acids produced by several *Penicillium* species, cloning the gene involved in the production of an unknown dihydroxy fatty acid according to sequence analysis of putative fatty acid diol synthase genes, identifying hydroxy fatty acids produced from unsaturated fatty acids by the expressed enzyme, and determining kinetic parameters for unsaturated fatty acids.

## MATERIALS AND METHODS

### Materials

Fatty acid standards, including palmitoleic acid, oleic acid, linoleic acid, conjugated linoleic acid, α-linolenic acid, γ-linolenic acid, eicosadienoic acid, dihomo-γ-linolenic acid, arachidonic acid, docosapentanoic acid, and docosahexaenoic acid, were purchased from Santa Cruz Biotechnology. Samples of 5*S*,8*R*-DiHODE, 8*R*-HODE, and 8*R*-HPODE were prepared from linoleic acid by the reactions of recombinant *Escherichia coli* cells expressing *A. nidulans* 5*S*,8*R*-diol synthase and its H1004A-C1006S variant (19). 7*S*,8*S*-DiHODE and 8*R*,11*S*-DiHODE were prepared from linoleic acid using recombinant *E. coli* cells expressing fatty acid diol synthase from *Glomerella cingulate* (unpublished observations) and crude enzyme extract from *A. clavatus* (5), respectively. The converted oxylipins were processed to >99% purity by a previously described method (20) and subsequently used as reference standards. Enantiomeric mixtures [8-^2^H]8-HODE and [8,11-^2^H_2_]8,11-DiHODE were prepared by oxidation with Dess-Martin periodinane and reduction with NaB^2^H_4_ as described previously (7).

### Crude enzyme preparation

*P*. *chrysogenum* KACC 41892, *Penicillium digitatum* KCCM 60140, *Penicillium oxalicum* KCTC 6440, *Penicillium marneffei* KCCM 60287, and *A. clavatus* KCCM 60329 were used as the sources of crude enzymes to identify putative hydroxy linoleic acids produced from linoleic acid. Fungal spores were incubated on potato dextrose agar plates at 28°C for 5 days. Four agar pieces (10 × 10 mm) from the plate were used to inoculate a 100 ml baffled flask containing 25 ml of potato dextrose broth and 5 mM linoleic acid to induce protein expression, with shaking at 150 rpm for 5 days. Mycelia were harvested by filtration, washed three times with saline solution, and homogenized on ice for 10 min in 50 mM HEPES buffer (pH 7.5). Mycelial debris and unbroken mycelia were removed by centrifugation at 13,000 *g* for 30 min at 4°C, and the supernatant was filtered through a 0.45 μm filter. The filtrates were then used as crude *Penicillium* enzyme extracts.

### Cloning and site-directed mutagenesis

*P. chrysogenum* and *E. coli* ER2566 were used as the sources of DNA template for fatty acid diol synthase gene and host cells, respectively. pET-21a(+) plasmid, which contained nucleotides encoding six histidine residues at C-terminal position, was used as an expression vector. Total RNA was isolated from the mycelia of *P. chrysogenum* using a Hybrid-R total RNA purification kit (GeneAll). Full-length cDNA was synthesized from total RNA by reverse transcription PCR using a reverse transcription kit (TaKaRa). The gene encoding fatty acid diol synthase and the full and partial genes of N-terminal domain or C-terminal domain were cloned using the Gibson assembly method (21). The sequences of the primers used for gene cloning were based on the DNA sequence of *P. chrysogenum* fatty acid diol synthase (GenBank accession number CAP97986). Primers were designed to amplify the expression vector and fatty acid diol synthase DNA fragments in supplementary Table 1. The amplified DNA fragments and linearized vector, which were obtained by PCR with Phusion High-Fidelity DNA Polymerase (New England Biolabs), were ligated using Gibson Assembly Master Mix (New England Biolabs). *E. coli* strain ER2566 was transformed with the ligation mixture and plated on Luria-Bertani (LB) agar containing 20 μg/ml ampicillin. An ampicillin-resistant colony was selected, and plasmid DNA from the transformant was isolated using a plasmid purification kit (Intron). Site-directed mutagenesis was performed using a QuikChange kit, according to the manufacturer’s protocol (Stratagene).

### Purified enzyme preparation

Recombinant *E. coli* cells were cultivated in a 2 liter flask containing 500 ml of LB medium and 20 μg/ml ampicillin at 37°C with shaking at 200 rpm. When the optical density at 600 nm of the bacterial culture reached 0.6, isopropyl-β-d-thiogalactopyranoside was added to a final concentration of 0.1 mM to induce enzyme expression. The culture was then incubated at 16°C with shaking at 150 rpm for 16 h to express the enzyme. Cells from the culture broth were harvested by centrifugation at 10,000 *g* for 30 min at 4°C and washed three times with 0.85% saline solution. The washed recombinant cells were subsequently resuspended in 50 mM phosphate buffer (pH 7.0) containing 300 mM NaCl and 1 mg/ml lysozyme. The resuspended cells were disrupted by sonication on ice for 10 min. The unbroken cells and cell debris were removed by centrifugation at 13,000 *g* for 20 min at 4°C, and the supernatant was passed through a 0.45 μm filter. The filtrate was applied to a HisTrap HP affinity chromatography column (GE Healthcare) equilibrated with 50 mM phosphate buffer (pH 7.0) on a fast-protein liquid chromatography (Bio-Rad) in a cold room at 4°C. The bound protein was eluted at 4°C using the same buffer containing 250 mM imidazole at a flow rate of 1 ml/min. The active fractions were collected and dialyzed against 50 mM 3-[4-(2-hydroxyethyl)-1-piperazinyl]-propane sulfonic acid (EPPS) buffer (pH 8.0) at 4°C for 16 h. After dialysis, the resulting solution was used as the purified enzyme. 

### Enzyme assays

Unless otherwise stated, the reactions were performed at 25°C for 5 min in 50 mM EPPS buffer (pH 8.0) containing 0.5 mM unsaturated fatty acid and 50 μg/ml purified enzyme or 5 mg/ml crude enzyme extract. The effect of pH and temperature on the activity of *P. chrysogenum* 8*R*,11*S*-LDS toward linoleic acid was investigated by varying the pH from 5.5 to 9.0, using 50 mM MES buffer (pH 5.5−6.0), 50 mM HEPES buffer (pH 6.0−8.0), 50 mM EPPS buffer (pH 8.0−8.5), and 50 mM 2-(cyclohexylamino)ethanesulfonic acid buffer (pH 8.6−9.0) at 25°C and by varying the temperature from 5°C to 65°C at pH 6.5. The effect of temperature on enzyme stability was investigated by varying the temperature from 15°C to 55°C in 50 mM HEPES buffer (pH 6.5). Enzyme activity was determined after incubating the solution at each temperature for 2 h. To determine the kinetic parameters of the DOX activity in 8*R*,11*S*-LDS, O_2_ uptake was measured by a Clark-type 5300A biological oxygen monitor (YSI) with 1 μg/ml purified enzyme and unsaturated fatty acids such as palmitoleic acid, oleic acid, linoleic acid, and α-linolenic acid (12.5 μM to 1 mM). The concentrations of linoleic acid and α-linolenic acid as substrates in the range of 50 to 500 μM were used to determine the kinetic parameters of whole enzyme using an HPLC system (Agilent 1100). The kinetic parameters, *K_m_* and *k_cat_*, were determined using a Hanes-Woolf plot based on the Michaelis-Menten equation.

### Structural analysis

Homology modeling of C-terminal domains of 8*R*,11*S*-LDS from *P. chrysogenum* and 5*S*,8*R*-LDS from *A. nidulans* was performed using Build Homology Models module in the MODELER application of Discovery Studio (DS) 4.0 (Accerlys) based on the crystal structure of allene oxide synthase (AOS) from guayule [Protein Data Bank (PDB) entry 3DBM] as a template. Comparative modeling was used to generate the most probable structure of the query protein by aligning it with the template sequence, simultaneously considering spatial restraints, and local molecular geometry. The generated structure was improved by subsequent refinement of the loop conformations by assessing the compatibility of amino acid sequences with the known PDB structures using Protein Health module in DS 4.0. The geometry of the loop region was corrected using the Refine Loop/MODELER, and the best model was selected. The quality of the model was analyzed by PROCHECK (22). Hydrogen atoms were added to the model and minimized to have a stable energy conformation and to relax the conformation from close contacts. 8*R*-HPODE as a substrate was docked into the active-site pocket in the model of C-terminal domains of 8*R*,11*S*-LDS from *P. chrysogenum* and 5*S*,8*R*-LDS from *A. nidulans* using C-DOCKER module, and a sphere with a radius of 4.5 Å around the ligand-binding pocket of the enzyme was defined as the active site. Candidate poses were created using random rigid-body rotations, followed by simulated annealing. The structures of the protein, substrate, and their complexes were subjected to energy minimization using the CHARMM force field in DS 4.0 (23). Full-potential final minimization was used to refine the substrate poses. The energy-docked conformation of the substrate was retrieved for postdocking analysis using C-DOCKER module. The substrate orientation giving the lowest interaction energy was chosen for subsequent rounds of docking.

### Analytical methods

The reaction products were extracted using an equal volume of ethyl acetate, the solvent was then removed with a rotary evaporator, and methanol was added to the dried extracts. Fatty acids and oxylipins were quantitatively analyzed using the HPLC system mentioned previously, with a UV detector at a detection wavelength of 202 nm and a reversed-phase Nucleosil C_18_ column (3.2 × 150 mm, 5 μm particle size; Phenomenex). The column was eluted at 35°C with a gradient of solvent A (acetonitrile-water-acetic acid, 50:50:0.1, v/v/v) and solvent B (acetonitrile-acetic acid, 100:0.1, v/v) as follows: 100% solvent A at a flow rate of 0.25 ml/min for 0–5 min; solvent A to solvent B for 5− 21 min at 0.25 ml/min; for 21–22 min at 0.4 ml/min; 100% solvent B at 0.4 ml/min for 22–27 min; solvent B to solvent A at 0.4 ml/min for 27–32 min; and 100% solvent A at 0.25 ml/min for 32–35 min. Chiral phase (CP)-HPLC and normal phase (NP)-HPLC were run using Chiralcel OD-H column (2.1 × 150 mm, 5 μm particle size; Daicel) and Zorbax RX SIL column (2.1 × 150 mm, 5 μm particle size; Agilent) with solvent systems of *n*-hexane/2-propanol/acetic acid (88:12:0.1, v/v/v) and *n*-hexane/diethyl ether/acetic acid (70:30:0.25), respectively.

LC/MS/MS analysis of oxylipins was performed using a Thermo-Finnigan LCQ Deca XP Plus ion trap mass spectrometer (Thermo Scientific). The instrument consisted of an LC pump, an autosampler, and a photodiode array detector. Ionization of the samples was carried out using ESI. The operation parameters were as follows: capillary temperature, 275°C; ion source voltage, 5 kV; nebulizer gas, 206.84 kPa; capillary voltage, 46 V in positive mode and 15 V in negative ionization mode; average scan time, 0.6 s; average time to change polarity, 1.2 s; and collision energy, ∼35% abundance of the precursor ion.

## RESULTS

### Assessment of the hydroxy fatty acids produced from linoleic acid by crude *Penicillium* enzyme extracts

**Fig. 1.**
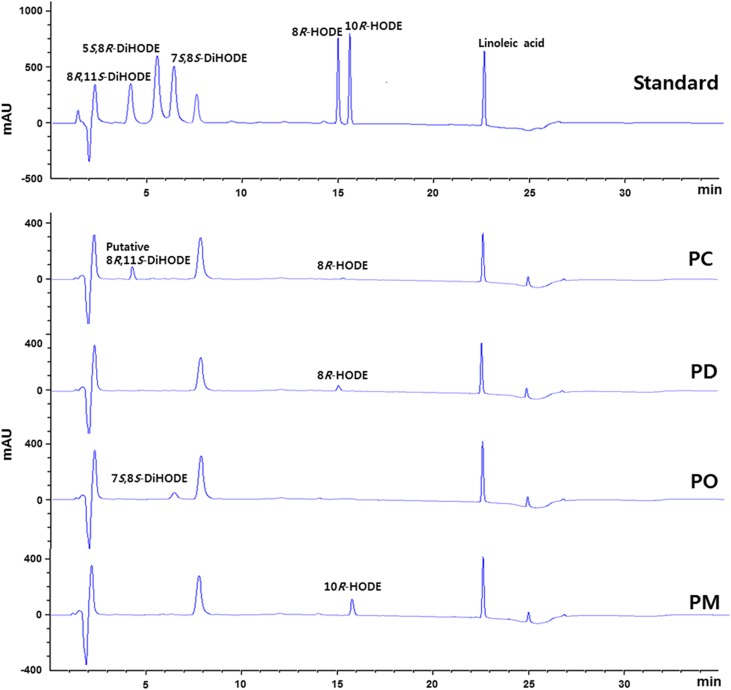
HPLC profiles for the conversions of linoleic acid to putative hydroxy fatty acids by crude enzyme extracts from *P*. *chrysogenum* (PC), *P*. *digitatum* (PD), *P*. *oxalicum* (PO), and *P*. *marneffei* (PM). Compounds used as standards, including 5*S*,8*R*-DiHODE, 7*S*,8*S*-DiHODE, 8*R*,11*S*-DiHODE, 8*R*-HODE, 10*R*-HODE, and linoleic acid, are shown. Crude *P. chrysogenum* (PC_1) enzyme extract produced a putative 8*R*,11*S*-DiHODE. The peak at about 7.7 min represents EPPS buffer.

Crude enzyme extracts from *P. chrysogenum*, *P. digitatum*, *P. marneffei*, and *P. oxalicum* containing putative diol synthases were used for the conversion of linoleic acid to hydroxy fatty acids. The reaction products were analyzed by HPLC using a reversed phase Nucleosil C_18_ column and reference standards, including 5*S*,8*R*-DiHODE, 7*S*,8*S*-DiHODE, 8*R*,11*S*-DiHODE, 8*R*-HODE, and 10*R*-HODE. Crude enzyme extracts from *P. digitatum, P. oxalicum*, and *P. marneffei* converted linoleic acid to products, with the same retention times as those of 8*R*-HODE, 7*S*,8*S*-DiHODE, and 10*R*-HODE, respectively (**Fig. 1**). However, the retention time (4.3 min) of the compound produced by crude *P. chrysogenum* enzyme extract was the same as that of 8*R*,11*S*-DiHODE, suggesting that the compound was 8,11-DiHODE.

### Amino acid sequence analysis of putative diol synthases from *Penicillium* species

**Fig. 2.**
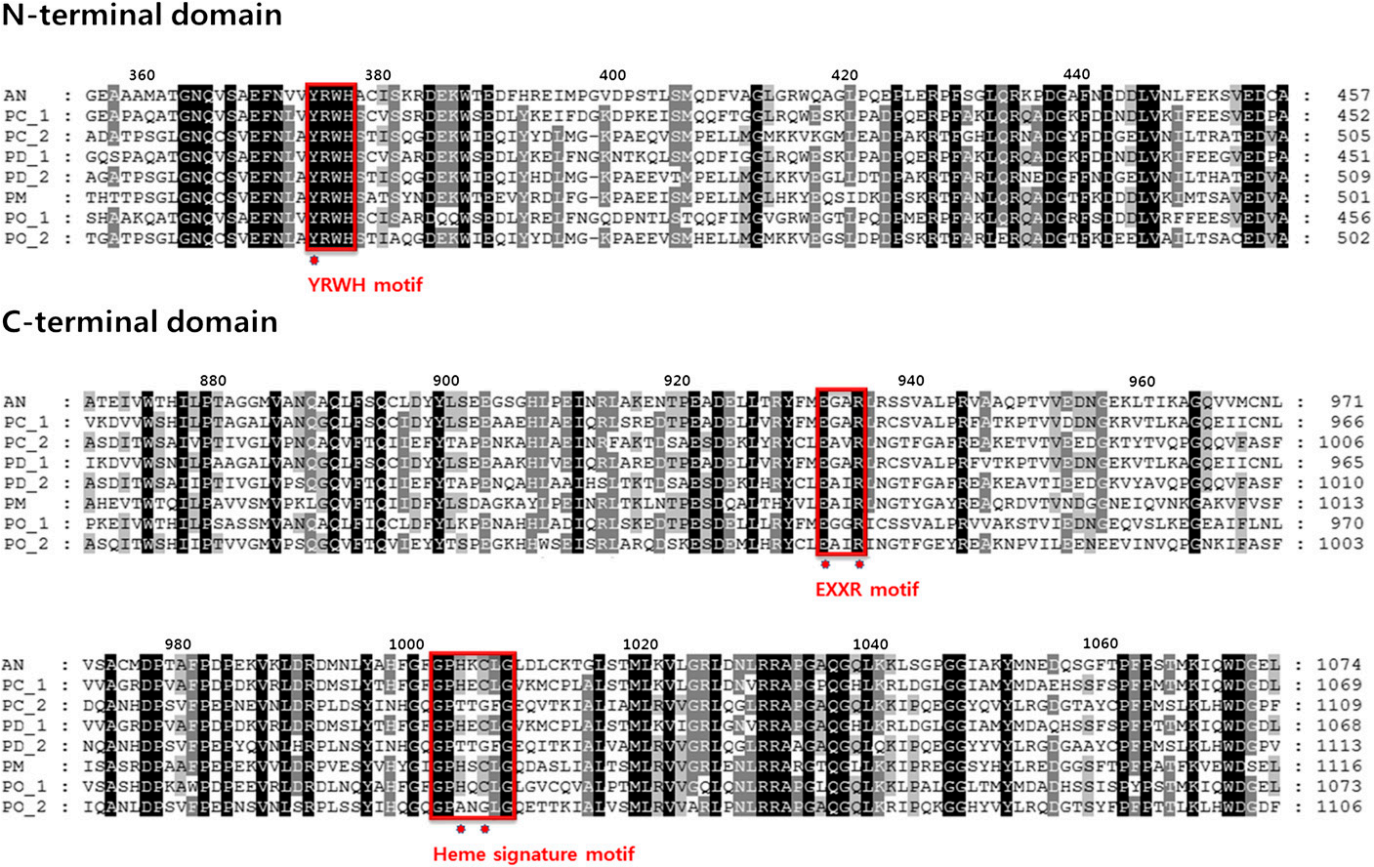
Alignment of partial amino acid sequences of N- and C-terminal domains in putative diol synthase. Putative diol synthase sequences from *P*. *chrysogenum* (PC_1 and PC_2), *P*. *digitatum* (PD_1 and PD_2), *P*. *marneffei* (PM), and *P*. *oxalicum* (PO_1 and PO_2), and the sequence of PpoA from *A*. *nidulans* (AN) were aligned using ClustalW2. YRWH, EXXR, and heme signature motifs and crucial amino acids for the activities of N- and C-terminal domains in the motifs are represented by the red boxes and red dots, respectively. The GenBank accession numbers are as follows: AN, AAR88626; PC_1, CAP97986; PC_2, CAP94248; PD_1, EKV17116; PD_2, EKV06981; PM, EEA26582; PO_1, EPS31749; PO_2, EPS29986.

The production of monohydroxy and dihydroxy linoleic acids from linoleic acid by *Penicillium* species resulted from the reactions of the corresponding putative fatty acid diol synthases in these species. To analyze such enzymes, amino acid sequences of putative fatty acid diol synthases from *Penicillium* species, including PC_1 and PC_2 from *P. chrysogenum*, PD_1 and PD_2 from *P. digitatum*, PM from *P. marneffei*, and PO_1 and PO_2 from *P. oxalicum*, were aligned with that of PpoA from *A. nidulans* (**Fig. 2**). The YRWH motif of the N-terminal domain containing the catalytic residue Tyr^374^ of *A. nidulans* PpoA (2) and the Glu and Arg residues in EXXR motif of the C-terminal domain were found to be conserved across all of the *Penicillium* candidate proteins. However, the two crucial residues for the hydroperoxide isomerase activity in the C-terminal heme signature motif, His^1004^ and Cys^1006^ of *A. nidulans* PpoA, were conserved in *P. chrysogenum* PC_1, *P. digitatum* PD_1, *P. marneffei* PM, and *P. oxalicum* PO_1, but not in *P. chrysogenum* PC_2, *P. digitatum* PD_2, and *P. oxalicum* PO_2 (Fig. 2). These results suggest that PC_1, PD_1, PM, and PO_1 convert linoleic acid to dihydroxy linoleic acids, whereas PC_2, PD_2, and PO_2 convert linoleic acid to monohydroxy linoleic acids. As *P. chrysogenum* converted linoleic acid to putative 8,11-DiHODE, the gene encoding PC_1 was cloned, with the aim of identifying a novel 8,11-diol synthase.

### Gene cloning and purification of the putative fatty acid diol synthase from *P*. *chrysogenum*

A 3,225 bp gene encoding PC_1 from *P. chrysogenum* (1,074 amino acids), with the same sequence as that in GenBank (accession number CAP97986) (supplementary Fig. 1), and the full and partial genes encoding the N-terminal or C-terminal domain (supplementary Fig. 2) were cloned and expressed in *E. coli*, respectively. The N-terminal domain of the putative fatty acid diol synthase from *P*. *chrysogenum* (supplementary Fig. 3A) was identified as the amino acid residues of 1−669 by sequence alignment (supplementary Fig. 3B) with that of 5*S*,8*R*-LDS from *A. fumigatus* (4), which was the amino acid residues of 1−674 (supplementary Fig. 3C). The expressed protein of the residues of 1−669 was active, whereas the expressed protein of the residues of 93−642, which was predicted as the N-terminal peroxidase-like superfamily by NCBI, exhibited no activity for unsaturated fatty acids. Thus, the C-terminal domain was the amino acid residues of 670−1,074 as the rest of the enzyme except for the N-terminal domain; however, it exhibited no activity for 8*R*-HPODE.

The expressed protein exhibited the catalytic residue Tyr^369^ in its N-terminal domain and the crucial residues for hydroperoxide isomerase activity such as His^999^ and Cys^1001^ in its C-terminal domain. These residues were critical for the conversion of unsaturated fatty acid to dihydroxy fatty acid. The recombinant enzyme was purified as a soluble protein from crude *E. coli* extract by HisTrap affinity chromatography. The putative fatty acid diol synthase from *P. chrysogenum* was purified with a final purification of 6.4-fold, a yield of 21.2% compared with crude enzyme, and the specific activity of the purified enzyme for the conversion of linoleic acid to dihydroxy fatty acid was 1.43 μmol/min/mg.

### Identification of the products obtained from the conversion of unsaturated fatty acids by the putative fatty acid diol synthase from *P*. *chrysogenum*

**Fig. 3.**
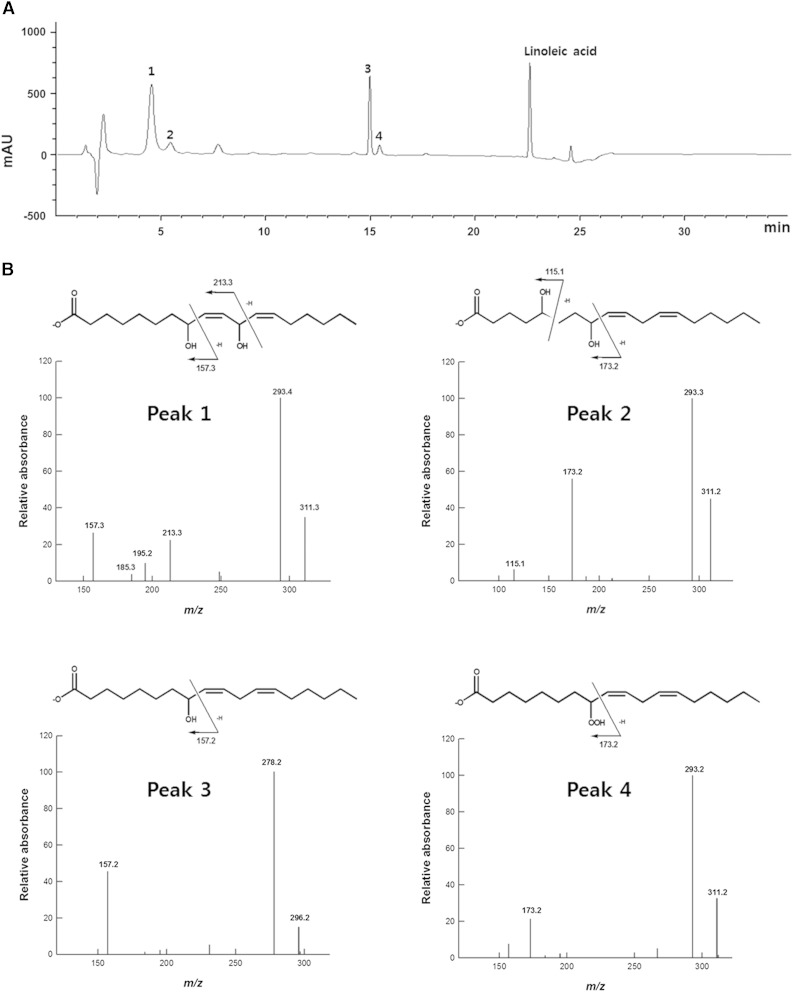
HPLC and LC/MS/MS analyses of the products obtained from the conversion of linoleic acid by the putative diol synthase from *P*. *chrysogenum*. A: HPLC profile of the products obtained from the conversion of linoleic acid. Unknown peaks 1, 2, 3, and 4 were assumed to be oxylipins. B: LC/MS/MS spectra and chemical structures of the products resulting from the conversion of linoleic acid. Peaks 1, 2, 3, and 4 were identified as 8,11-DiHODE, 5,8-DiHODE, 8-HODE, and 8-HPODE, respectively. The peak at ∼7.7 min represents EPPS buffer.

The products obtained from the conversion of linoleic acid by the putative fatty acid diol synthase from *P. chrysogenum* are represented in the HPLC profile given in **Fig. 3A**. Unknown peaks 1, 2, 3, and 4, which were assumed to be oxylipins, were analyzed by LC/MS/MS (Fig. 3B). The molecular anions of the products were represented by *m*/*z* 311 for peak 1 (4.3 min retention time in HPLC), *m*/*z* 311 for peak 2 (5.5 min), *m*/*z* 296 for peak 3 (14.9 min), and *m*/*z* 311 for peak 4 (15.3 min). Data regarding the fragments formed by cleavage of hydroxylated fatty acids have been reported previously (24). The *m*/*z* 157 and 213 for peak 1, *m*/*z* 115 and 173 for peak 2, *m*/*z* 157 for peak 3, and *m*/*z* 173 for peak 4 resulted from α-cleavage of the hydroxyl groups at the C8 and C11 positions, the C5 and C8 positions, and the C8 position, and the hydroperoxyl group at the C8 position, respectively. The *m*/*z* 293 for peak 1, *m*/*z* 293 for peak 2, *m*/*z* 278 for peak 3, and *m*/*z* 293 for peak 4 were derived from the loss of water from molecular anion of each product. Based on this analysis, the compounds represented by peaks 1, 2, 3, and 4 were identified as 8,11-DiHODE, 5,8-DiHODE, 8-HODE, and 8-HPODE, respectively. The LC/MS/MS spectrum of peak 1 consisted of the same pattern as that of the previously reported 8*R*,11*S*-DiHODE (7). 8,11-DiHODE in the HPLC profile was a major dihydroxy fatty acid product, whereas 5,8-DiHODE was a minor reaction product (Fig. 3A), suggesting that the enzyme was 8,11-diol synthase.

**Fig. 4.**
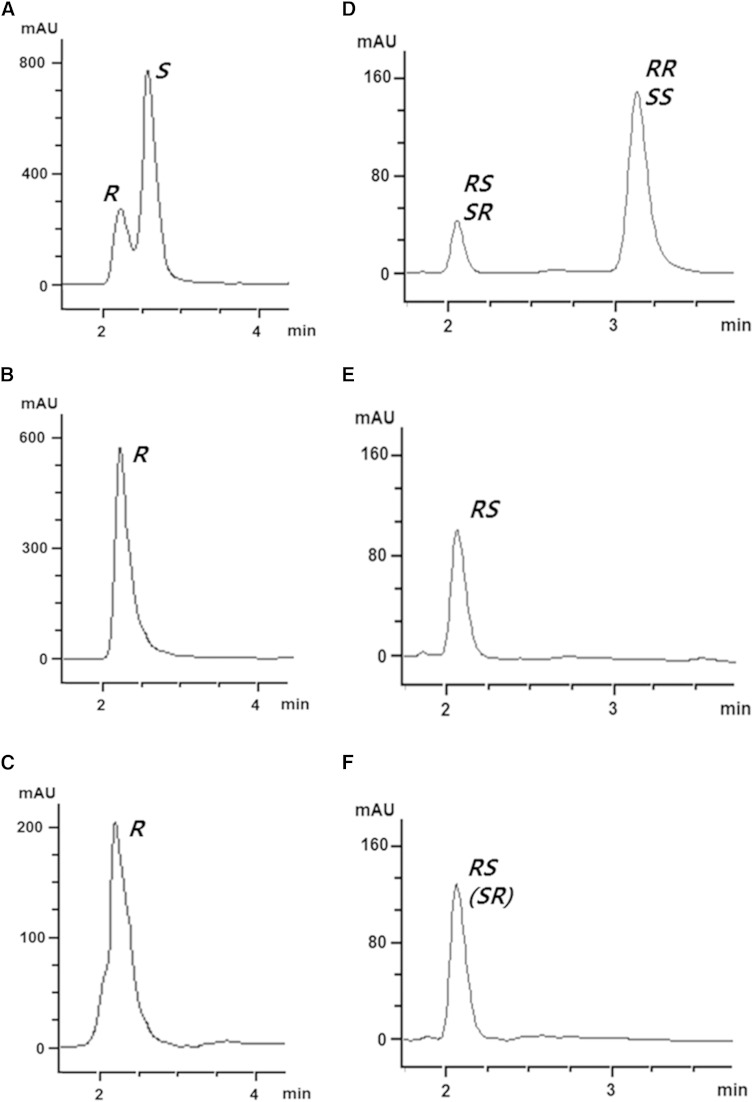
Steric analysis of 8-HODE and 8,11-DiHODE. CP-HPLC and NP-HPLC were run using Chiralcel OD-H column (2.1 × 150 mm, 5 μm particle size; Daicel) with *n*-hexane/2-propanol/acetic acid (88:12:0.1, v/v/v) and Zorbax RX SIL column (2.1 × 150 mm, 5 μm particle size; Agilent) with *n*-hexane/diethyl ether/acetic acid (70:30:0.25), respectively. A: CP-HPLC profile of enantiomeric mixture [8-^2^H]8-HODE. B: CP-HPLC analysis of 8*R*-HODE, which was prepared by H1004A-C1006S variant 5,8-LDS from *A. nidulans*. C: CP-HPLC profile of 8-HODE, which was converted by H999A-C1001S variant 8,11-LDS from *P. chrysogenum*. D: NP-HPLC profile of diastereoisomers of enantiomeric mixture [8,11-^2^H_2_]8,11-DiHODE. E: NP-HPLC profile of 8*R*,11*S*-DiHODE, which was prepared by crude enzyme extract from *A. clavatus*. F: NP-HPLC analysis of 8,11-DiHODE, which was produced by the putative diol synthase from *P. chrysogenum*.

Enantiomeric mixtures [8-^2^H]8-HODE and [8,11-^2^H_2_]8,11-DiHODE were prepared to determine the stereoconfigurations. The chirality of 8-HODE produced by the putative fatty acid diol synthase from *P. chrysogenum* was confirmed by CP-HPLC with 8*R*-HODE produced by the H1004A-C1006S variant of 5*S*,8*R*-LDS from *A. nidulans* (19) and enantiomeric mixture [8-^2^H]8-HODE. The retention time of the earlier eluent of enantiomeric mixture [8-^2^H]8-HODE (**Fig. 4A**) was the same as those of 8*R*-HODE obtained from the H1004A-C1006S variant (Fig. 4B) and 8-HODE produced by the putative fatty acid diol synthase (Fig. 4C). Therefore, the later eluent of enantiomeric mixture [8-^2^H]8-HODE was 8*S*-HODE, and the product of the enzyme was 8*R*-HODE. The diastereoisomers of enantiomeric mixture [8,11-^2^H_2_]8,11-DiHODE were separated as two eluents by NP-HPLC. The retention time of the earlier eluent of enantiomeric mixture [8,11-^2^H_2_]8,11-DiHODE (Fig. 4D) was the same as those of 8*R*,11*S*-DiHODE (Fig. 4E), which was prepared by crude enzyme extract from *A. clavatus*, and 8,11-DiHODE, which was produced from linoleic acid by the putative fatty acid diol synthase from *P. chrysogenum* (Fig. 4F). The results indicated that the later eluent of enantiomeric mixture [8,11-^2^H_2_]8,11-DiHODE was 8*R*,11*R*- or 8*S*,11*S*-DiHODE and that 8,11-DiHODE was 8*R*,11*S*- or 8*S*,11*R*-DiHODE. Because 8*R*-HPODE produced by the putative fatty acid diol synthase from *P. chrysogenum* was a precursor of 8*R*,11*S*-DiHODE, the produced 8,11-DiHODE was 8*R*,11*S*-DiHODE.

**Fig. 5.**
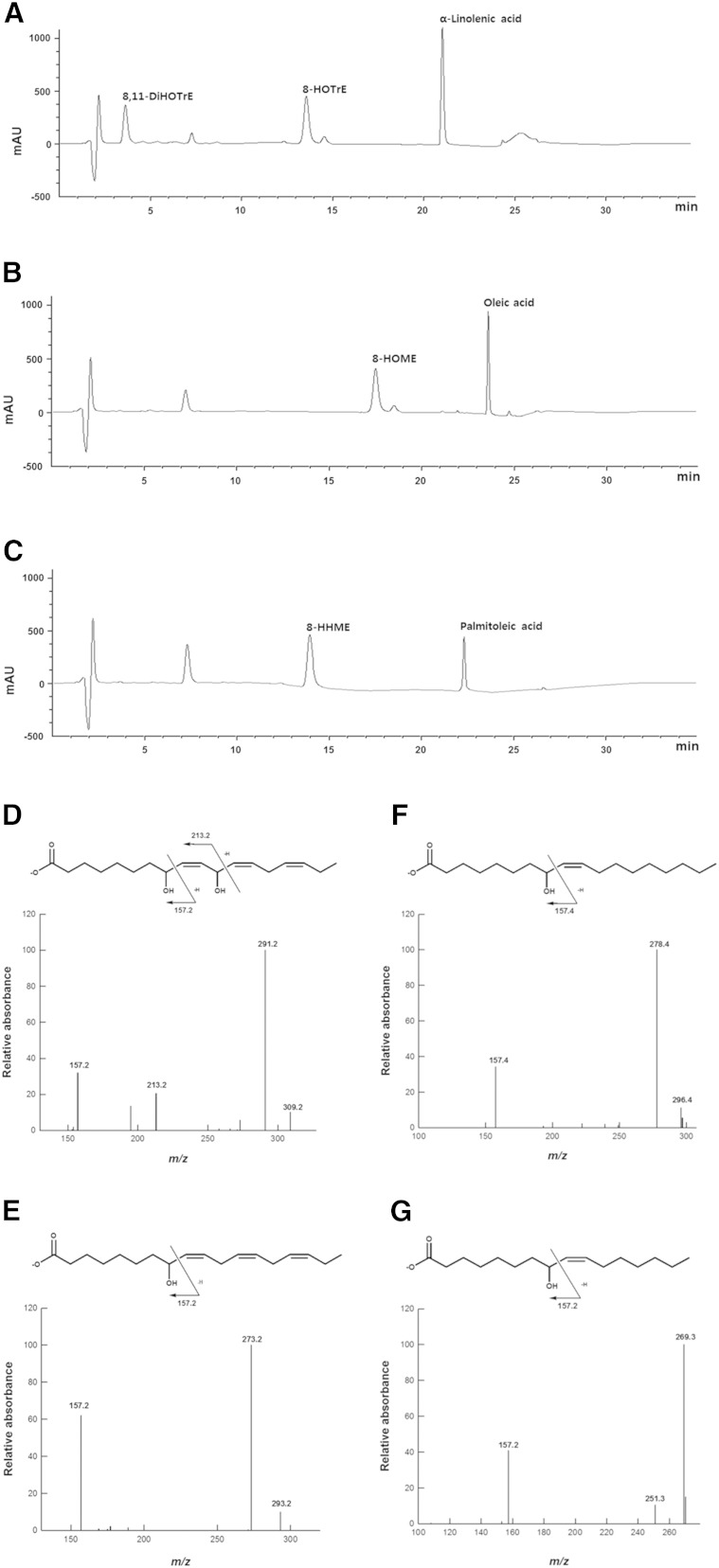
HPLC and LC/MS/MS analyses of the products obtained from the conversions of α-linolenic acid, oleic acid, and palmitoleic acid by the putative diol synthase from *P*. *chrysogenum*. HPLC profiles of the products obtained from the conversion of α-linolenic acid (A), oleic acid (B), and palmitoleic acid (C). LC/MS/MS spectra and chemical structures of 8,11-DiHOTrE (D), 8-HOTrE (E), 8-HOME (F), and 8-HHME (G) obtained from the conversion of α-linolenic acid, oleic acid, and palmitoleic acid. The peak at ∼7.7 min represents EPPS buffer.

The products obtained from the conversion of α-linolenic acid, oleic acid, and palmitoleic acid by *P. chrysogenum* 8*R*,11*S*-diol synthase are shown in the HPLC profiles (**Fig. 5A–C**, respectively). The molecular anion of the compound produced from α-linolenic acid by the enzyme was represented by a peak at *m*/*z* 309, while peaks at *m*/*z* 157 and 213 resulted from cleavage of the hydroxyl groups at the C8 and C11 positions, respectively (Fig. 5D). These peaks represent the compound as 8,11-dihydroxy-9,12,15(*Z*,*Z*,*Z*)-octadecatrienoic acid (8,11-DiHOTrE). The molecular anion of the products obtained from the conversion of α-linolenic acid, oleic acid, and palmitoleic acid were represented by peaks at *m*/*z* 293 (Fig. 5E), 296 (Fig. 5F), and 269 (Fig. 5G), respectively. A peak at *m*/*z* 157 in each profile resulted from cleavage of the hydroxyl group at the C8 position for each product. Thus, these compounds were identified as 8-hydroxy-9,12,15(*Z*,*Z*,*Z*)-​octadecatrienoic acid (8-HOTrE), 8-hydroxy-9(*Z*)-octadecenoic acid (8-HOME), and 8-hydroxy-9(*Z*)-hexadecenoic acid (8-HHME), respectively. 

### Biochemical properties of 8*R*,11*S*-diol synthase from *P*. *chrysogenum*

The expressed protein was visualized by SDS-PAGE as a single band of ∼120 kDa (supplementary Fig. 1), which was consistent with the calculated value of 120,998 Da based on the 1,074 amino acid residues plus 6 histidine residues. Truncated N-terminal and C-terminal domains were expressed in cell debris as the bands of ∼74 and 45 kDa, respectively, in SDS-PAGE. However, the C-terminal domain protein was not observed as soluble enzyme in crude extract, and thus it was not purified by HisTrap affinity chromatography (supplementary Fig. 2). The activities of truncated N-terminal domain for linoleic acid and truncated C-terminal domain for 8*R*-HPODE were evaluated by whole cells containing each domain protein or crude enzyme extracts. Truncated N-terminal domain exhibited activity, whereas truncated C-terminal domain showed no activity. However, the activity of truncated N-terminal domain reduced to 60% of the DOX activity of whole enzyme. Therefore, the DOX activity of whole enzyme was measured to determine the activity of N-terminal domain.

Heme occupancy of the wild-type and H999A, C1001S, and H999A-C1001S variant enzymes of 8*R*,11*S*-diol synthase from *P*. *chrysogenum* was determined by measuring the ratio of heme absorption to protein absorption (*A*_410_/*A*_280_) with a UV-visible spectroscopy (supplementary Fig. 4). The H999A, C1001S, and H999A-C1001S variant enzymes showed 0.8-, 0.6-, 0.4-fold lower heme occupancy than the wild-type enzyme, respectively. The hydroperoxide isomerase activity of the double-site H999A-C1001S variant was completely abolished, whereas those of the H999A and C1001S variants remained. The ratios of heme absorption to protein absorption of the wild-type and variant enzymes were closely related with the hydroperoxide isomerase activities.

The effect of pH on the enzymatic production of 8*R*-HODE or 8*R*,11*S*-DiHODE from linoleic acid was investigated at 25°C. The maximum rates of 8*R*-HODE production by DOX activity and 8*R*,11*S*-DiHODE production by whole enzyme were observed at pH 8.0 and 6.5, respectively (supplementary Fig. 5). The effect of temperature on enzyme activity was assessed at pH 6.5, and the maximum activities of both DOX and whole enzyme were observed at 25°C (supplementary Fig. 6). The thermal stability of the enzyme was examined by measuring activity after incubation for 2 h at temperatures ranging from 15°C to 55°C. As temperature increased, 8*R*,11*S*-DiHODE production by whole enzyme decreased and stopped at 55°C (supplementary Fig. 7), indicating that the activity of C-terminal domain was completely abolished. However, 8 *R*-HODE production by DOX activity was exhibited below 65°C. Thus, the N-terminal domain demonstrated greater thermal stability than the C-terminal domain.

The production of monohydroxy fatty acids from substrates by the enzyme followed the order α-linolenic acid > linoleic acid > palmitoleic acid > oleic acid (**Table 1**). However, no activity was observed for conjugated linoleic acid, γ-linolenic acid, eicosadienoic acid, dihomo-γ-linolenic acid, arachidonic acid, docosapentaenoic acid, and docosahexaenoic acid. Dihydroxy fatty acids were only produced by *P. chrysogenum* 8*R*,11*S*-diol synthase when α-linolenic acid and linoleic acid were used as substrates, and the specific production using linoleic acid was higher than that using α-linolenic acid. The Michaelis-Menten constants (*K_m_*), turnover numbers (*k_cat_*), and catalytic efficiencies (*k_cat_*/*K_m_*) of *P*. *chrysogenum* 8*R*,11*S*-diol synthase for unsaturated fatty acids are presented in **Table 2**. The *k_cat_* order of DOX activity for unsaturated fatty acids (α-linolenic acid > linoleic acid > palmitoleic acid > oleic acid) was the same as that observed for its specific activity for the production of monohydroxy fatty acids. However, *k_cat_*/*K_m_* values of DOX activity were in the order linoleic acid > palmitoleic acid > oleic acid > α-linolenic acid, while the order of *K_m_* of DOX activity was exactly the reverse order of *k_cat_*/*K_m_*. The catalytic efficiency of whole enzyme for linoleic acid was higher than that for α-linolenic acid.

**TABLE 1.** Specific activity of *P*. *chrysogenum* 8*R*,11*S*-diol synthase toward unsaturated fatty acids

		Specific Production (μmol/min/mg)	
Substrate	Product	Monohydroxy Fatty Acid	Dihydroxy Fatty Acid
Palmitoleic acid (16:1^Δ9^*^Z^*)	8-HHME	9.6 ± 0.2	NA
Oleic acid (18:1^Δ9^*^Z^*)	8-HOME	9.0 ± 0.1	NA
Linoleic acid (18:2^Δ9^*^Z^*^,12^*^Z^*)	8-HODE, 8,11-DiHODE	10.8 ± 0.1	1.4 ± 0.0
Conjugated linoleic acid (18:2^Δ9^*^E^*^,12^*^E^*)	NA	NA	NA
α-Linolenic acid (18:3^Δ9^*^Z^*^,12^*^Z^*^,15^*^Z^*)	8-HOTrE, 8,11-DiHOTrE	14.9 ± 0.0	1.1 ± 0.0
γ-Linolenic acid (18:3^Δ6^*^Z^*^,9^*^Z^*^,12^*^Z^*)	NA	NA	NA
Eicosadienoic acid (20:2^Δ11^*^Z^*^,14^*^Z^*)	NA	NA	NA
Dihomo-γ-linolenic acid (20:3^Δ8^*^Z^*^,11^*^Z^*^,14^*^Z^*)	NA	NA	NA
Arachidonic acid (20:4^Δ5^*^Z^*^,8^*^Z^*^,11^*^Z^*^,14^*^Z^*)	NA	NA	NA
Docosapentaenoic acid (22:5^Δ^*^7Z^*^,10^*^Z^*^,13^*^Z^*^,16^*^Z^*^,119^*^Z^*)	NA	NA	NA
Docosahexaenoic acid (22:6^Δ4^*^Z^*^,7^*^Z^*^,10^*^Z^*^,13^*^Z^*^,16^*^Z^*^,19^*^Z^*)	NA	NA	NA

NA, no activity.

**TABLE 2.** Kinetic parameters of *P*. *chrysogenum* 8*R*,11*S*-diol synthase toward unsaturated fatty acids

	DOX Activity			Whole Enzyme Activity*^a^*		
Substrate	*K_m_* (μM)	*k_cat_* (min^−1^)	*k_cat_*/*K_m_* (μM^−1^ min^−1^)	*K_m_* (μM)	*k_cat_* (min^−1^)	*k_cat_*/*K_m_* (μM^−1^ min^−1^)
Palmitoleic acid	20.0 ± 0.5	4,910 ± 226	259	NA	NA	NA
Oleic acid	34.3 ± 1.2	4,528 ± 56	134	NA	NA	NA
Linoleic acid	18.9 ± 0.6	5,392 ± 151	285	86.1 ± 1.8	622 ± 7.2	7.2
α-Linolenic acid	69.9 ± 0.3	7,907 ± 113	113	138 ± 3.2	829 ± 16.8	6.0

NA, no activity.

a The activity for the conversion of fatty acid to dihydroxy fatty acid by the sequential reactions of N-terminal DOX domain and C-terminal hydroperoxide isomerase domain.

## DISCUSSION

The C-terminal crucial residues for the hydroperoxide isomerase activity (His^1004^ and Cys^1006^) of *A. nidulans* 5,8-LDS (PpoA) (2) were conserved across *P. chrysogenum* PC_1, *P. digitatum* PD_1, *P. marneffei* PM, and *P. oxalicum* PO_1 but differed from those of *P. chrysogenum* PC_2, *P. digitatum* PD_2, and *P. oxalicum* PO_2 (Fig. 2). These results suggest that PC_1, PD_1, PM, and PO_1 can convert linoleic acid to dihydroxy linoleic acids, while PC_2, PD_2, and PO_2 are only able to convert it to monohydroxy linoleic acids. The crude enzyme extracts from *P. digitatum* and *P. oxalicum* converted linoleic acid to a monohydroxy linoleic acid (8*R*-HODE) and a dihydroxy linoleic acid (7*S*,8*S*-DiHODE), respectively. Thus, *P. digitatum* PD_2 and *P. oxalicum* PO_1 may be involved in this activity. Although *P. marneffei* PM containing the crucial residues His and Cys for hydroperoxide isomerase activity was expected to produce a dihydroxy linoleic acid, the enzyme produced monohydroxy linoleic acid (10*R*-HODE), indicating that the His and Cys residues in the C-terminal domain may not be critical for 10*R*-DOX activity. We proceeded to clone the gene *P. chrysogenum* PC_1 containing one catalytic residue for DOX activity (Tyr^369^) and two crucial residues for hydroperoxide isomerase activity (His^999^ and Cys^1001^) because PC_1 might be responsible for the production of the putative 8,11-DiHODE, which was produced without 10*R*-HODE accumulation by crude *P. chrysogenum* enzyme extract (Fig. 1).

The products obtained from the conversion of linoleic acid by the putative fatty acid diol synthase from *P*. *chrysogenum* were identified by LC/MS/MS as 8,11-DiHODE, 5,8-DiHODE, 8-HODE, and 8-HPODE (Fig. 3), and the 8,11-DiHODE and 8-HODE products were identified as 8*R*,11*S*-DiHODE and 8*R*-HODE, respectively, by steric analysis. When a high concentration of the putative fatty acid diol synthase (5 mg/ml) was used to completely convert linoleic acid to dihydroxy linoleic acids for the exact evaluation of dihydroxy fatty acids, the enzyme produced 8*R*,11*S*-DiHODE (97% of DiHODE) as a main product with the by-product 5,8-DiHODE (supplementary Fig. 8), indicating that the enzyme was 8*R*,11*S*-diol synthase. We replaced the crucial residues for the hydroperoxide isomerase activity in the C-terminal domain of this enzyme, His^999^ and Cys^1001^, with alanine and serine, respectively. As a result, decrease in heme occupancies of the variant enzymes led to a decrease of their activities, and a double-site variant (H999A-C1001S) completely abolished hydroperoxide isomerase activity (supplementary Fig. 4). The double-site variant of *P. chrysogenum* 8*R*,11*S*-diol synthase produced only a monohydroperoxy fatty acid, 8*R*-HPODE, without producing dihydroxy fatty acids, in the same manner as that of the H1004A-C1006S variant of *A. nidulans* 5*S*,8*R*-diol synthase (19). The 8*R*-HPODE produced was converted to 8*R*-HODE by the addition of cysteine as a reducing agent (supplementary Fig. 9). These results demonstrated that 8*R*,11*S*-diol synthase from *P*. *chrysogenum* converted linoleic acid to 8*R*,11*S*-DiHODE via 8*R*-HPODE and shared a common mechanism with other fatty acid diol synthases (5). Among the substrates tested, both DOX activity and whole enzyme activity exhibited the highest catalytic efficiency for linoleic acid (Table 2). Therefore, this enzyme is characterized as an 8*R*,11*S*-LDS.

*P. chrysogenum* 8*R*,11*S*-LDS converted α-linolenic acid, linoleic acid, palmitoleic acid, and oleic acid to the monohydroxy fatty acids 8-HOTrE, 8-HODE, 8-HHME, and 8-HOME, respectively. Moreover, it produced the dihydroxy fatty acids 8*R*,11*S*-DiHODE and 8,11-DiHOTrE from linoleic acid and α-linolenic acid, respectively (Table 1). Based on this activity, we observed that unsaturated fatty acids in which the first *cis* double bond was located at the C9 and C10 positions were converted to hydroxy fatty acids. Unsaturated fatty acids containing one double bond and two or more double bonds were converted to monohydroxy fatty acids and dihydroxy fatty acids via monohydroxy fatty acids, respectively. Whether monohydroxy or dihydroxy fatty acids are formed by *P. chrysogenum* 8*R*,11*S*-LDS may therefore depend on the position and number of carbon-carbon double bonds in the unsaturated fatty acid substrate.

Some fungi such as *A. fumigatus*, *A. niger*, and *A. clavatus* convert linoleic acid to the by-product 8*R*,11*S*-DiHODE as well as 5*S*,8*R*-DiHODE (5, 8, 12); however, the enzymes involved in 8*R*,11*S*-DiHODE production have not been identified. It was previously assumed that PpoB was responsible for 8*R*,11*S*-diol synthase activity in *A. fumigatus*. However, the biosynthesis of 8*R*,11*S*-DiHODE by *A. fumigatus* has only been observed in cytosolic fractions of *A. fumigatus*, and the enzyme involved in 8*R*,11*S*-DiHODE biosynthesis (PpoB) is not found (12). Thus, the 8,11-LDS isolated from *P. chrysogenum* in the present study constitutes a novel fatty acid diol synthase.

**Fig. 6.**
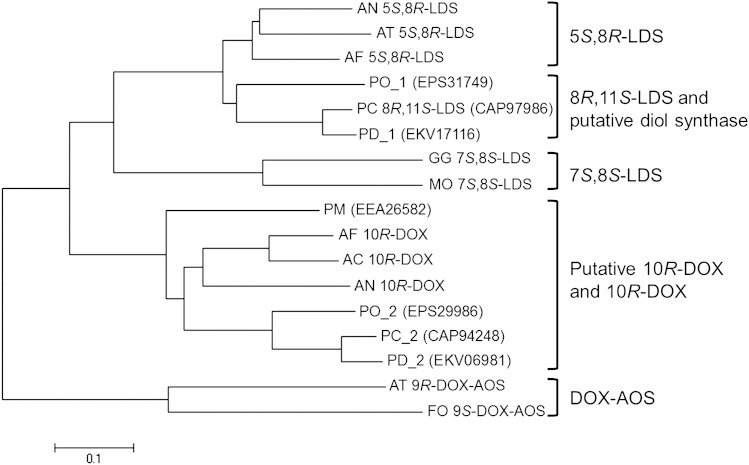
Phylogenetic tree based on amino acid sequences of diol synthase-related fungal proteins. The tree was constructed using MEGA6, based on the full amino acid sequences of selected proteins from five groups. The bar below the tree represents an estimation of the replacement rate per residue. The putative diol synthase group may constitute an 8*R*,11*S*-LDS group. Characterized proteins are indicated by the following enzyme names: 5*S*,8*R*-LDS, 8*R*,11*S*-LDS, 7S,8S-LDS, 10*R*-DOX, 9*R*-DOX-AOS, and 9*S*-DOX-AOS. GenBank accession numbers are as follows: AN 5*S*,8*R*-LDS, EAA65132; AT 5*S*,8*R*-LDS, AGA95448; AF 5*S*,8*R*-LDS, EDP50447; PC 8*R*,11*S*-LDS, CAP97986; GG 7*S*,8*S*-LDS, AAD49559; MO 7*S*,8*S*-LDS, EHA52010; AF 10*R*-DOX, ABV21633; AC 10*R*-DOX, EAW09782; AN 10*R*-DOX, AAT36614; AT 9*R*-DOX-AOS, AGH14485; FO 9*S*-DOX-AOS, EGU88194. AN, *Aspergillus nidulans*; AT, *Aspergillus terreus*; AF, *Aspergillus fumigatus*; PO, *Penicillium oxalicum*; PC, *Penicillium chrysogenum*; PD, *Penicillium digitatum*; GG, *Gaeumannomyces graminis*; MO, *Magnaporthe oryzae*; PM, *Penicillium marneffei*; AC, *Aspergillus clavatus*; FO, *Fusarium oxysporum*.

Amino acid sequences of fatty acid diol synthase-related fungal proteins, including that of 8*R*,11*S*-LDS from *P. chrysogenum*, were used to construct a phylogenetic tree (**Fig. 6**). Five groups were recovered, with each protein being categorized as a 5*S*,8*R*-LDS (2, 4, 9), 7*S*,8*S*-LDS (25, 26), 10*R*-DOX (3–5), DOX-AOS (9, 27), or 8*R*,11*S*-LDS and putative fatty acid diol synthase. *P. marneffei* PM, *P. oxalicum* PO_2, *P. chrysogenum* PC_2, and *P. digitatum* PD_2 clustered together in the putative 10*R*-DOX and 10*R*-DOX group. The putative fatty acid diol synthase group containing *P. chrysogenum* 8*R*,11*S*-LDS was more closely related to the LDS groups than those containing the 10*R*-DOX and DOX-AOS enzymes, with the 5*S*,8*R*-LDS group being its nearest phylogenetic neighbor. The amino acid sequence of 8*R*,11*S*-LDS from *P. chrysogenum* exhibited 74, 70, and 69% identity with 5*S*,8*R*-LDSs from *A. fumigatus*, *A. nidulans*, and *A. terreus*, respectively; 45% and 44% identity with 7*S*,8*S*-LDSs from *Magnaporthe oryzae* and *Gaeumannomyces graminis*, respectively; 44, 44, and 43% identity with 5*S*,8*R*-LDSs from *A. fumigatus*, *A. clavatus*, and *A. nidulans*, respectively; and 35% and 33% identity with DOX-AOSs from *A. terreus* and *Fusarium oxysporum*, respectively. Sequence analysis indicated that 8*R*,11*S*-LDS from *P. chrysogenum* is closest to 5*S*,8*R*-LDS from *A. fumigatus*.

The homology models for the active-site in the C-terminal domains of *P. chrysogenum* 8*R*,11*S*-LDS and *A. nidulans* 5*S*,8*R*-LDS were generated based on the known structure of AOS from guayule (PDB entry 3DBM) as a template. A ligand docking study with 8*R*-HPODE was conducted using these homology models to identify the specific residues involved in substrate binding. The proposed active-site residues within a sphere of 4.5 Å radius near to the docked 8*R*-HPODE with the heme of the C-terminal domain of *P. chrysogenum* 8*R*,11*S*-LDS were identified as Lys^729^, Val^738^, Ala^739^, Val^744^, Arg^839^, Glu^905^, Arg^908^, and Phe^976^, and those of *A. nidulans* 5*S*,8*R*-LDS were Lys^734^, Lys^738^, Glu^910^, and Phe^981^ (supplementary Fig. 10). Among the proposed active-site residues, the different residue between the two enzymes was Val^738^ in *P. chrysogenum* 8*R*,11*S*-LDS and Lys^738^ in *A. nidulans* 5*S*,8*R*-LDS. These residues may be potential amino acids to be determinant of substrate specificity. However, determination of the protein structure with the substrate bound as well as site-directed mutagenesis experiments involving the active-site residues predicted to be involved in substrate binding are necessary to provide further evidence of this conclusion because of low amino acid identity with the template structure.

## CONCLUSION

A putative fatty acid diol synthase gene from *P. chrysogenum* was cloned and expressed, and the expressed protein was characterized. The enzyme was found to be a novel 8*R*,11*S*-LDS by identifying its products and determining its substrate specificity. Discovery of this novel fatty acid diol synthase may provide additional information on the difference with other-type fatty acid diol synthases and reproduction of *Penicillium* species. Discovery of this novel fatty acid diol synthase may assist in the characterization of other enzymes and cast light on reproduction in *Penicillium* species.

## Supplementary Material

Supplemental Data
