# Supplementary material for: Characterization of a novel 8R,11S-linoleate diol synthase from Penicillium chrysogenum by identification of its enzymatic products
Source: J Lipid Res. 2016 Feb;57(2):207–18. doi: 10.1194/jlr.M061341 (PMC4727417; doi:10.1194/jlr.M061341)
Supplement: Supplemental Data [file supp_57_2_207__index.html]

Characterization of a novel 8R,11S-linoleate diol synthase from Penicillium chrysogenum by identification of its enzymatic products — Characterization of a novel 8R,11S-linoleate diol synthase from Penicillium chrysogenum by identification of its enzymatic products — Supplemental Data 

# Characterization of a novel 8*R*,11*S*-linoleate diol synthase from *Penicillium chrysogenum* by identification of its enzymatic products

## Supplemental Data

- Table S1, Fig. S1, Fig. S2, Fig. S3, Fig. S4, Fig. S4, Fig. S5, Fig. S6, Fig. S7, Fig. S8, Fig. S9, and Fig. S10 (.docx, 2.1 MB) - Table S1. Primers for cloning of the gene and partial genes encoding diol synthase, N-terminal domain, and C-terminal domain and site-directed mutagenesis (SDM) of H999A, C1001S, and H999A- C1001S Fig. S1. SDS-PAGE analysis of P. chrysogenum 8R,11S-LDS at each purification step. Fig. S2. SDS-PAGE analysis of N-terminal and C-terminal domains of 8R,11S-LDS from P. chrysogenum at each purification step. Fig. S3. (A) N-Terminal heme peroxidase (dioxygenase) and C-terminal cytochrome P450-heme thiolate (hydroperoxide isomerase) domains of putative diol synthase from P. chrysogenum predicted by NCBI. (B) Alignment of partial amino acid sequences of N-terminal domains of the putative diol synthase from P. chrysogenum with 5S,8R-LDS from A. fumigatus. Fig. S4. Absorbance of wild type and variant enzymes of 8R,11S-LDS from P. chrysogenum. Fig. S5. Effect of pH on the activity of P. chrysogenum 8R,11S-LDS. Fig. S6. Effect of temperature on the activity of P. chrysogenum 8R,11S-LDS. Fig. S7. Effect of temperature on the stability of P. chrysogenum 8R,11S-LDS. Fig. S8. HPLC analysis identifying the major 8R,11S-DiHODE and minor 5S,8R-DiHODE products obtained from linoleic acid by a high concentration of P. chrysogenum 8R,11S-LDS. Fig. S9. HPLC analysis of the conversion of linoleic acid to 8R-HPODE and 8R-HODE by the H999A-C1001S variant of P. chrysogenum 8R,11S-LDS. Fig. S10. Docking poses for 8R-HPODE interacting with homology models of 8R,11S-LDS from P. chrysogenum and 5S,8R-LDS from A. nidulans.
