# Supplementary material for: Characterization of a novel 8R,11S-linoleate diol synthase from Penicillium chrysogenum by identification of its enzymatic products
Source: J Lipid Res. 2016 Feb;57(2):207–18. doi: 10.1194/jlr.M061341 (PMC4727417; doi:10.1194/jlr.M061341)
Supplement: Supplemental Data [file 10.1194_M061341_jlr.M061341-1.docx]

**Supplemental Material**

**Table S1.** Primers for cloning of the gene and partial genes encoding diol synthase, N-terminal domain, and C-terminal domain and site-directed mutagenesis (SDM) of H999A, C1001S, and H999A- C1001S

| Cloning or SDM | | Primer |
| --- | --- | --- |
| Cloning |  |  |
| Diol synthase | Insert | F: 5ʹ-AAGAAGGAGATATACATATGATGGCTGAGAAAGAGTCCAACTCC-3ʹ |
|  |  | R: 5ʹ-TCGAGTGCGGCCGCAAGCTTCTCCCTCCTGGCAGGCAGGTCT-3ʹ |
|  | Vector | F: 5ʹ-ACCTGCCTGCCAGGAGGGAGAAGCTTGCGGCCGCACTCGAGC-3ʹ |
|  |  | R: 5ʹ-TTGGACTCTTTCTCAGCCATATGTATATCTCCTTCTTAAAGTTAAA-3ʹ |
| N-terminal domain | Insert | F: 5ʹ-AAGAAGGAGATATACATATGGCTGAGAAAGAGTC-3ʹ |
|  |  | R: 5ʹ-AGTGCGGCCGCAAGCTTTACCTTGAAGCCTTCTT-3ʹ |
|  | Vector | F: 5ʹ-AAGAAGGCTTCAAGGTAAAGCTTGCGGCCGCACT-3ʹ |
|  |  | R: 5ʹ-GACTCTTTCTCAGCCATATGTATATCTCCTTCTT-3ʹ |
| C-terminal domain | Insert | F: 5ʹ-AAGGAGATATACATATGGTGTGGGGCGAGAAGAT-3ʹ |
|  |  | R: 5ʹ-AGTGCGGCCGCAAGCTTCTCCCTCCTGGCAGGCA-3ʹ |
|  | Vector | F: 5ʹ-TGCCTGCCAGGAGGGAGAAGCTTGCGGCCGCACT-3ʹ |
|  |  | R: 5ʹ-ATCTTCTCGCCCCACACCATATGTATATCTCCTT-3ʹ |
| Site-directed mutagenesis |  |  |
| H999A |  | F: 5ʹ-ttggcttcggtcccgctgaaagcctgggcg-3ʹ |
|  |  | R: 5ʹ-cgcccaggctttcagcgggaccgaagccaa-3ʹ |
| C1001S |  | F: 5ʹ-gcttcggtccccatgaaagcctgggcg-3ʹ |
|  |  | R: 5ʹ-cgcccaggctttcatggggaccgaagc-3ʹ |
| H999A-C1001S |  | F: 5'-attttcacgcccaggctttcagcgggaccgaagccaaag-3' |
|  |  | R: 5'-ctttggcttcggtcccgctgaaagcctgggcgtgaaaat-3' |

**Fig. S1.**


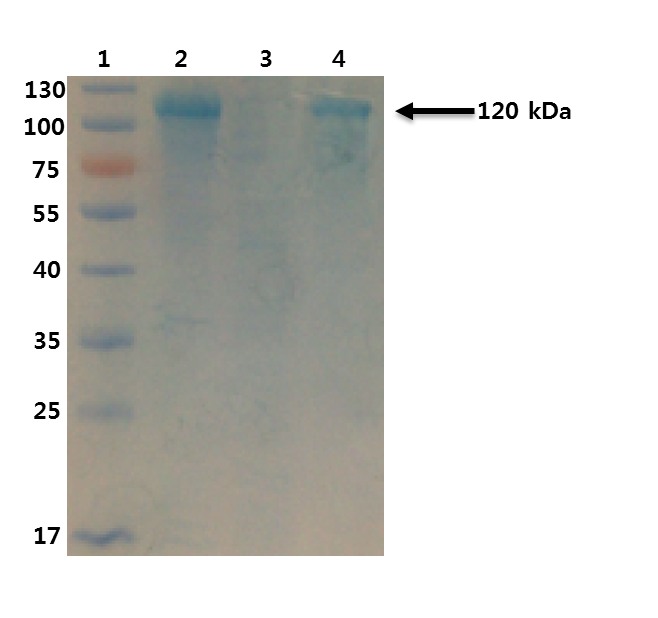


**Fig. S1.** SDS-PAGE analysis of *P. chrysogenum* 8*R*,11*S*-LDS at each purification step. *Lane 1*, molecular mass markers; *lane 2*, cell debris; *lane 3*, crude enzyme extract; *lane 4*, HisTrap HP column product (purified enzyme).

**Fig. S2.**


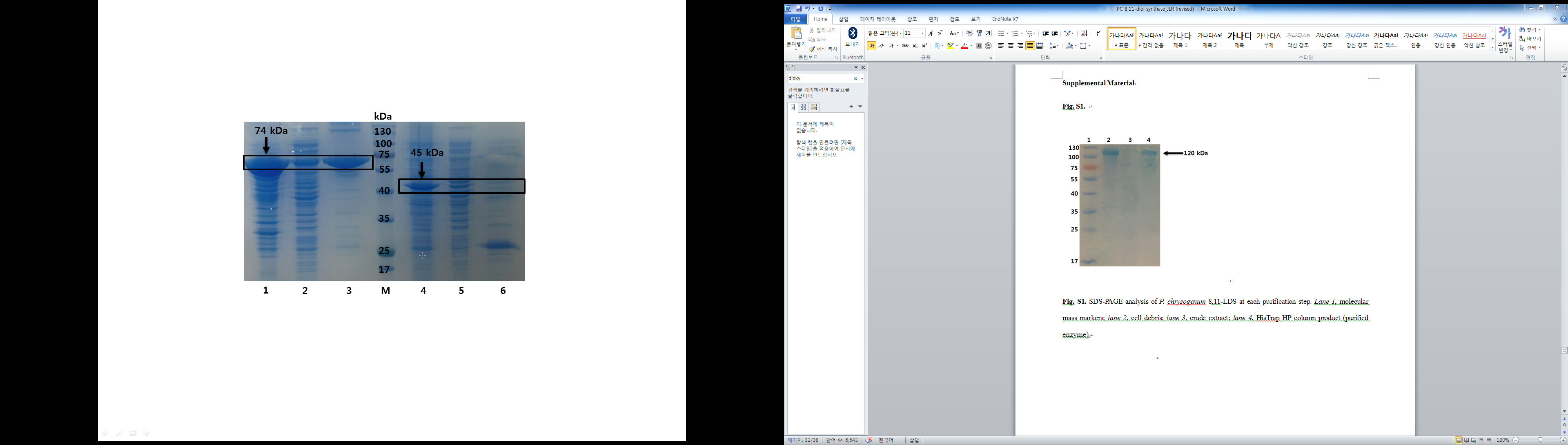


**Fig. S2.** SDS-PAGE analysis of N-terminal and C-terminal domains of 8*R*,11*S*-LDS from *P. chrysogenum* at each purification step. *Lane M*, molecular mass markers; *lane 1*, cell debris of N-terminal domain; *lane 2*, crude enzyme extract of N-terminal domain; *lane34*, HisTrap HP column product of N-terminal domain; *lane 1*, cell debris of C-terminal domain; *lane 2*, crude enzyme extract of C-terminal domain; *lane34*, HisTrap HP column product of C-terminal domain.

**Fig. S3.**

**A**


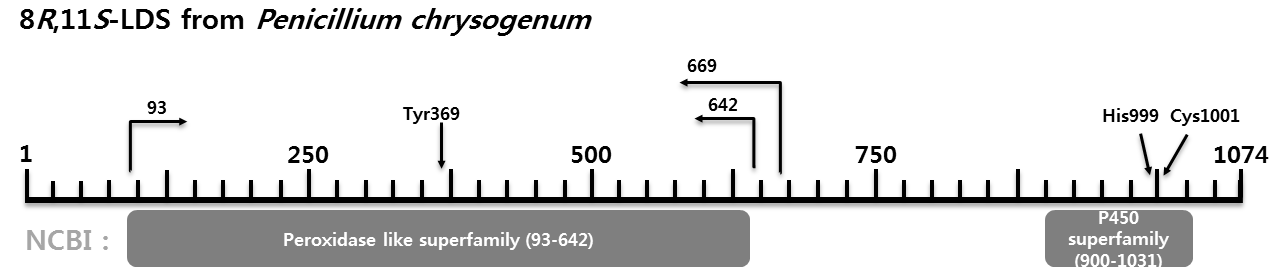


**B**


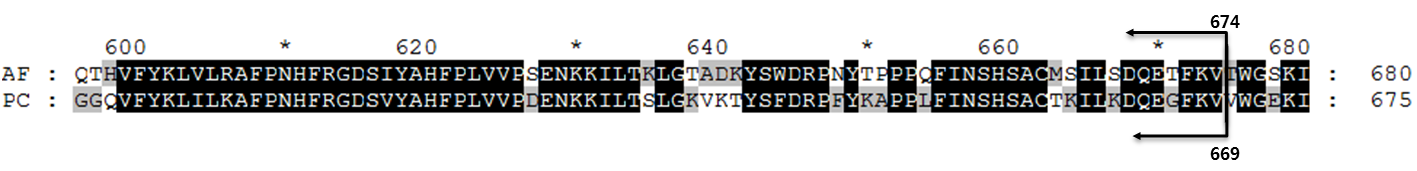


**C**


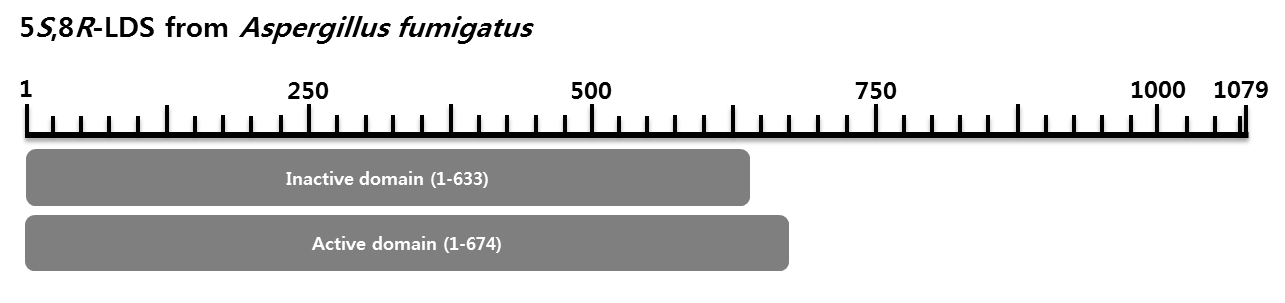


**Fig. S3.** (A) N-Terminal heme peroxidase (dioxygenase) and C-terminal cytochrome P450-heme thiolate (hydroperoxide isomerase) domains of putative diol synthase from *P. chrysogenum* predicted by NCBI. (B) Alignment of partial amino acid sequences of N-terminal domains of the putative diol synthase from *P. chrysogenum* with 5*S*,8*R*-LDS from *A. fumigatus*. Curved and straight arrows represent truncated protein sequences and critical residues for its activity, respectively. (C) Active and inactive N-terminal domains of 5*S*,8*R*-LDS from *A. fumigatus*.

**Fig. S4.**


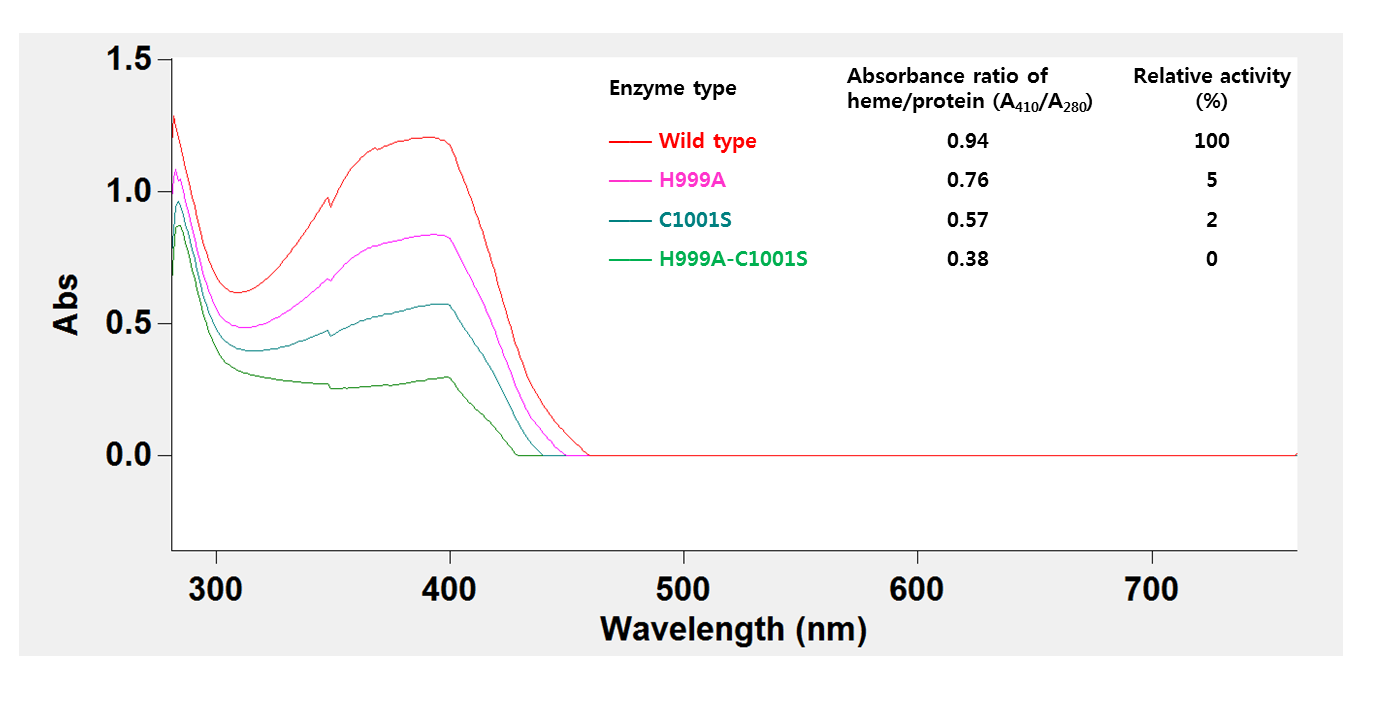


**Fig. S4.** Absorbance of wild type and variant enzymes of 8*R*,11*S*-LDS from *P. chrysogenum*. Spectra were recorded on a Carry 100 UV-vis spectrophotometer (Agilent, CA, USA) in 10mm cuvettes with 1 mg/ml enzymes. The table represents the ratio of heme absorbance to protein absorbance (A_410_/A_280_) and activity for conversion of linoleic acid to 8,11-DiHODE.

**Fig. S5.**

**A**

**

**B**

**Fig. S5.** Effect of pH on the activity of *P*. *chrysogenum* 8*R*,11*S*-LDS. (A) Effect of pH on the production of 8*R*,11*S*-DiHODE from linoleic acid. (B) Effect of pH on the production of 8*R*-HODE from linoleic acid. Data represent the means of three separate experiments and error bars represent standard deviations. Closed circles, open circles, closed squares, and open squares represent 50 mM MES buffer (pH 5.5−6.0), HEPES buffer (pH 6.0−8.0), EPPS buffer (pH 8.0−8.5), and CHES buffer (pH 8.6−9.0), respectively.

**Fig. S6.**

**A**

**B**

**Fig. S6.** Effect of temperature on the activity of *P*. *chrysogenum* 8*R*,11*S*-LDS. (A) Effect of temperature on the production of 8*R*,11*S*-DiHODE from linoleic acid. B, Effect of temperature on the production of 8*R*-HODE from linoleic acid. Data represent the means of three separate experiments and error bars represent standard deviations.

**Fig. S7.**

**Fig. S7.** Effect of temperature on the stability of *P*. *chrysogenum* 8*R*,11*S*-LDS. Data represent the means of three separate experiments and error bars represent standard deviations. Open circles, closed circles, and closed triangles represent 8*R*-HODE, 8*R*,11*S*-DiHODE, and 8*R*-HODE plus 8*R*,11*S*-DiHODE, respectively.

**Fig. S8.**


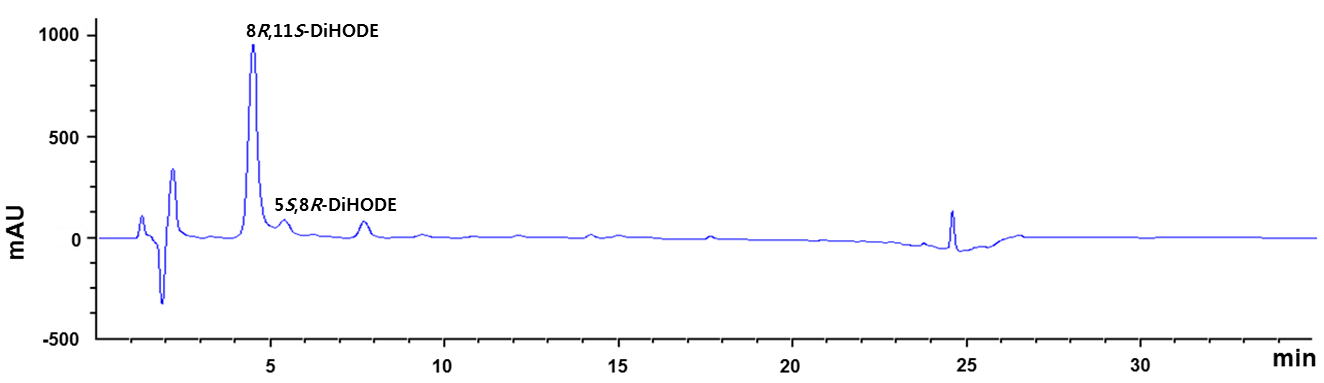


**Fig. S8.** HPLC analysis identifying the major 8*R*,11*S*-DiHODE and minor 5*S*,8*R*-DiHODE products obtained from linoleic acid by a high concentration of *P*. *chrysogenum* 8*R*,11*S*-LDS.

**Fig. S9.**


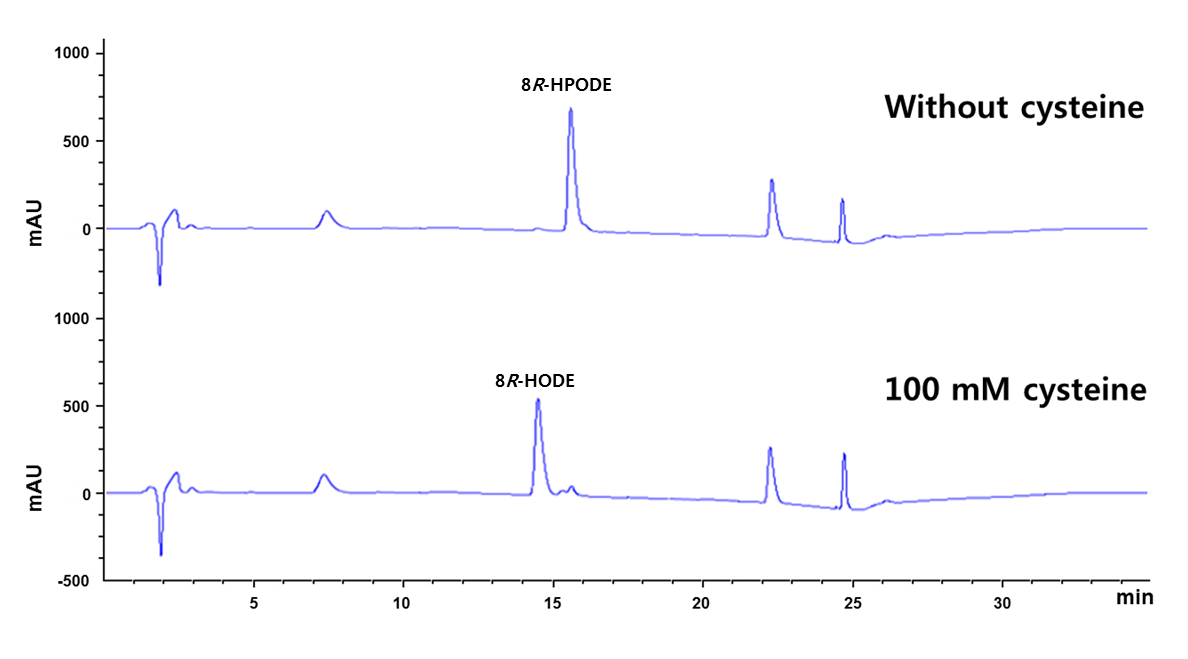


**Fig. S9.** HPLC analysis of the conversion of linoleic acid to 8*R*-HPODE and 8*R*-HODE by the H999A-C1001S variant of *P*. *chrysogenum* 8*R*,11*S*-LDS. The conversion of 8*R*-HPODE to 8*R*-HODE was achieved by the addition of cysteine to the reactant, as a reducing agent.

**Fig. S10.**

**A**


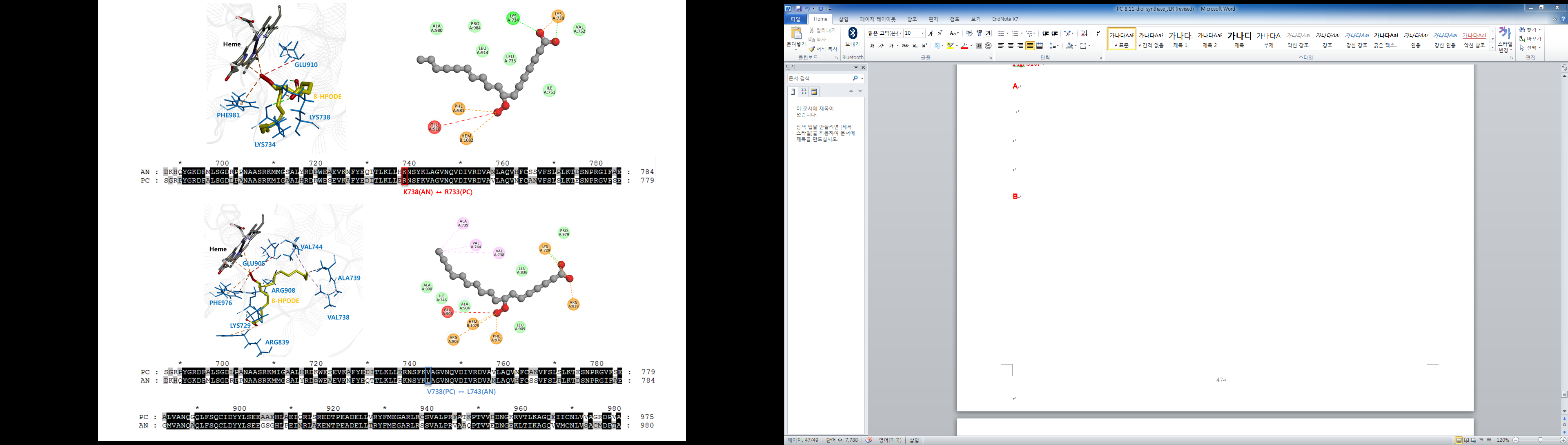


**B**


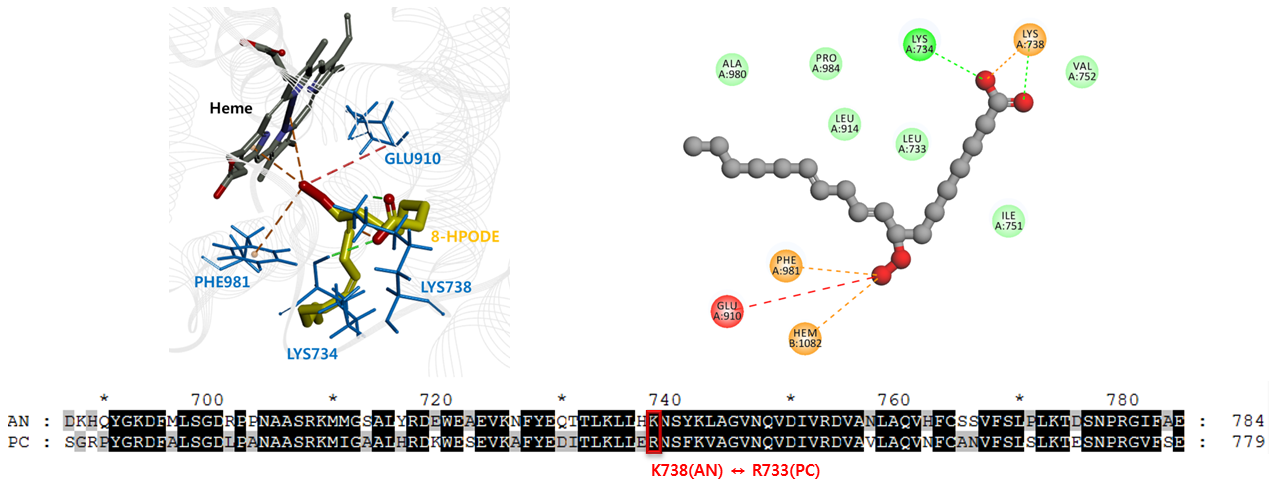


**Fig. S10.** Docking poses for 8*R*-HPODE interacting with homology models of 8*R*,11*S*-LDS from *P. chrysogenum* and 5*S*,8*R*-LDS from *A. nidulans*. (A) Docking pose of heme in C-terminal domain of 8*R*,11*S*-LDS from *P. chrysogenum* interacting with 8*R*-HPODE. The residues interacting with 8*R*-HPODE within a sphere of 4.5-Å radius centered on the substrate binding pocket around 8*R*-HPODE were Lys^729^, Val^738^, Ala^739^, Val^744^, Arg^839^, Glu^905^, Arg^908^, and Phe^976^. (B) Docking pose of heme in C-terminal domain of 5*S*,8*R*-LDS from *A. nidulans* interacting with 8*R*-HPODE. The residues interacting with 8*R*-HPODE within a sphere of 4.5-Å radius centered on the substrate binding pocket around 8*R*-HPODE were Lys^734^, Lys^738^, Glu^910^, and Phe^981^. Green, pink, orange, and red color dotted line or circle represent hydrogen bond, hydrophobic, electrostatic, and unfavorable negative-negative interactions, respectively. Blue and red boxes on the sequence alignment represent different residues between 8*R*,11*S*-LDS from *P. chrysogenum* and 5*S*,8*R*-LDS from *A. nidulans* among residues interacting with 8*R*-HPODE in docking poses for 8*R*-HPODE interacting with homology models of 8*R*,11*S*-LDS and 5*S*,8*R*-LDS, respectively. PC, 8*R*,11*S*-LDS from *P. chrysogenum*; AN, 5*S*,8*R*-LDS from *A. nidulans*.
